# What Parents of Children Who Have Received Emergency Care Think about Deferring Consent in Randomised Trials of Emergency Treatments: Postal Survey

**DOI:** 10.1371/journal.pone.0035982

**Published:** 2012-05-07

**Authors:** Carrol Gamble, Simon Nadel, Dee Snape, Andrew McKay, Helen Hickey, Paula Williamson, Linda Glennie, Claire Snowdon, Bridget Young

**Affiliations:** 1 Clinical Trials Research Centre, University of Liverpool, Liverpool, United Kingdom; 2 St Mary’s Hospital, Imperial College Healthcare NHS Trust, London, United Kingdom; 3 Mental and Behavioural Health Sciences, University of Liverpool, Liverpool, United Kingdom; 3 Meningitis Research Foundation, Thornbury, Bristol, United Kingdom; 4 London School of Hygiene and Tropical Medicine, London, United Kingdom; Nottingham University, United Kingdom

## Abstract

**Objective:**

To investigate parents’ views about deferred consent to inform management of trial disclosure after a child’s death.

**Methods:**

A postal questionnaire survey was sent to members of the Meningitis Research Foundation UK charity, whose child had suffered from bacterial meningitis or meningococcal septicaemia within the previous 5 years. Main outcome measures were acceptability of deferred consent; timing of requesting consent; and the management of disclosure of the trial after a child’s death.

**Results:**

220 families were sent questionnaires of whom 63 (29%) were bereaved. 68 families responded (31%), of whom 19 (28%) were bereaved. The majority (67%) was willing for their child to be involved in the trial without the trial being explained to them beforehand; 70% wanted to be informed about the trial as soon as their child’s condition had stabilised. In the event of a child’s death before the trial could be discussed the majority of bereaved parents (66% 12/18) anticipated wanting to be told about the trial at some time. This compared with 37% (18/49) of non-bereaved families (p = 0.06). Parents’ free text responses indicated that the word ‘trial’ held strongly negative connotations. A few parents regarded gaps in the evidence base about emergency treatments as indicating staff lacked expertise to care for a critically ill child. Bereaved parents’ free text responses indicated the importance of individualised management of disclosure about a trial following a child’s death.

**Discussion:**

Deferred consent is acceptable to the majority of respondents. Parents whose children had recovered differed in their views compared to bereaved parents. Most bereaved parents would want to be informed about the trial in the aftermath of a child’s death, although a minority strongly opposed such disclosure. Distinction should be drawn between the views of bereaved and non-bereaved parents when considering the acceptability of different consent processes.

## Introduction

When a child is admitted to hospital in an emergency situation his/her parents (or care providers) need to be able to draw some reassurance that the child is in the ‘best possible hands’ and receiving the ‘right treatment’. Yet many emergency treatments have crept in to common use without a robust evidence base [1]. Few clinicians or parents would disagree that clinical research in emergency, life-threatening conditions is required to improve patient outcomes; however there are many ethical issues that need to be addressed when designing a randomised controlled trial (RCT) to be conducted under these circumstances.

In 2004 the European Clinical Trials Directive [2], incorporating the ICH Harmonised Tripartite Guideline for Good Clinical Practice (ICH GCP) on clinical research was translated in to law across its member states [3]. This set valid informed consent as the cornerstone of experimental research involving human beings. However, the Directive made no provision for consent in emergency situations, thus creating a formidable barrier to research in this setting. Member states were forced either to operate at variance with the Directive or to accept restriction of such research. Internationally guidelines on emergency consent vary or are not specifically addressed [4]. The UK amended its legislation in 2006 to incorporate a deferred consent process in emergency situations for incapacitated adults [5] and in 2008 for minors [6] when the following conditions are met: (i) treatment is required urgently; (ii) urgent action is required for the purposes of the trial; (iii) it is not reasonably practicable to obtain consent prospectively; and (iv) an ethics committee has given approval to the procedure under which the action is taken.

The process of deferred consent allows patients to be included in research studies without obtaining their prior informed consent or that of their parent/carer (in the case of a legal minor), but requires that informed consent is obtained as soon as possible for continued study participation. In the US consent waivers are used, whereby researchers are not required to obtain patient consent at any stage provided that the research is granted ethical approval following public disclosure and community consultation [7]. In this context, community consultation may be regarded as a mandatory formalisation of patient and public involvement. However, the requirements for such consultation are unclear and this lack of guidance has been described as a barrier to emergency research in the US [8]. Deferred consent raises additional ethical dilemmas not present with consent waivers, such as: how and when to inform parents that their child has been included in a trial; how to handle the situation where a child dies before consent can be obtained but has been included in the trial; and the potential that the decision to decline is associated with a child’s poor outcome thereby creating bias in the trial results and conclusions [9]–[11].

Many treatments are routinely administered in children with life-threatening conditions infection, without a strong evidence base for their use [12]. Historically this is an extremely difficult situation in which to perform clinical trials given the considerable complexities involved in seeking parental consent in the emergency care setting, when children are critically ill and their parents are profoundly anxious and distressed. In this context, the method for seeking consent needs to avoid delaying patient treatment, whilst being as acceptable as possible to parents. Deferred consent has been approved to address these requirements, yet little is known about how parents view it or how to provide trial disclosure. We therefore carried out a survey with parents to explore their perspective on the proposed use of deferred consent in a double blind randomised controlled trial (RCT) which was being developed in the UK.

The RCT would compare the effectiveness of two treatments currently in widespread use and considered safe for children with presumed severe sepsis requiring emergency resuscitation and immediate treatment. In such a trial it may be harmful to delay a child’s treatment in order to seek prospective parental consent for the child’s involvement in the trial. To explore how to minimise the anxiety and distress that may be associated with involvement in a clinical trial in this intensely emotional setting, the views of parents who had first-hand experience of their child receiving emergency treatment for severe sepsis were investigated. Parents’ perceptions of deferred consent and opinions about how practitioners should make requests for deferred consent when a child had died before the trial could be discussed were sought.

## Methods

A postal survey was conducted to inform the design of a proposed double blind RCT within the UK.

The sample was drawn from a database of members of the Meningitis Research Foundation (MRF), a UK medical research charity which provides support to families affected by meningitis and septicaemia.

220 families were invited to take part in the survey that had a child admitted as an emergency within the last five years with bacterial meningitis (BM) or meningococcal septicaemia (MS). Had the proposed trial been running at the time of their child’s illness they would have received one of the treatments without their parents’ prior consent. Advice received from the UK Central Office for Research Ethics Committees was that ethical approval was not required for a survey of attitudes towards an RCT.

### Survey

The mailing included an invitation letter to explain the survey, a document describing the proposed trial emphasizing why the trial was needed, the difficulties of conducting a trial in this setting, and explaining that both treatments are in widespread use outside of the trial and two scenarios (appendix S1).

The two scenarios in the survey were set in the context of the proposed trial and its design:

Scenario A referred to a “child of mine” who needed emergency treatment. Because of the intensely sensitive nature of child death, we phrased Scenario B in more general terms, “If a child could not be resuscitated and unfortunately died in the emergency department…”.

A set of closed and open ended items followed the scenarios. The closed items in the survey had five response options ranging from strongly disagree to strongly agree.

The invitation letter, description of the proposed trial, scenarios and survey were developed by the study team, which included a lay person, clinician, psychologist, statistician, and trial manager. A decision was made in agreement with the MRF to accept no response to the initial contact as indicating unwillingness to take part.

### Analysis

The quantitative data from the closed items were analysed using simple descriptive statistics and the chi-square test for trend; exact tests were used as appropriate. Consistency of responses was considered by cross tabulations of responses between questions as an indication the survey was understood.

Analysis of the qualitative data, which comprised parents’ free text responses to the open ended items, drew on the principles of thematic analysis [13] and used both inductive and deductive approaches [14]. DS led the analysis, producing a coding frame that was iteratively developed to represent and code the data, as informed by BY’s and DS’s multiple readings of all free text responses and detailed discussions of the developing analysis. Procedural measures to ensure the quality of the analysis [15], [16] included attending to deviant cases and examining alternative formulations of the data. We documented the analysis, describing the key themes, sub-themes and areas of tension including extensive data extracts. Investigator triangulation involved PW, SN and CG reading and commenting upon this document to further refine and ‘test’ the analysis. The involvement of investigators with different perspectives in this process avoided any single perspective dominating and helped to connect the conclusions with practice and research. To evidence our interpretations verbatim excerpts from parents’ free text responses are presented accompanied with their identification numbers; ‘B’ indicates bereaved parents’ and ‘R’ indicates parents’ whose child had recovered. Square brackets […] signify omitted text while [text] indicates text that we added for clarification.

The results are presented according to overall themes within the survey grouped by whether the parent was bereaved or whether the child recovered. The qualitative findings are presented alongside those from the quantitative analysis to help contextualize and illuminate the quantitative responses.

## Results

Of the 220 families who were sent the postal survey 63 (29%) were bereaved. Sixty eight families responded, of which 19 (28%) were bereaved. The results grouped by bereavement status for scenario A, statements 1 to 4 are presented in Table 1 and scenario B statements 5 to 7 in Table 2. Sixty parents provided at least one free-text response (18 bereaved, 42 recovered).

**Table 1:** Scenario A: If a child of mine had a serious infection and needed emergency fluid treatment.

	Strongly disagree	Disagree	Neither agree nor disagree	Agree	Strongly agree	p-value
*Statement 1:* I would **not want** my child to be included in a clinical trial of these two commonly used fluids under any circumstances.						
Bereaved	7 (40)	9 (50)	1 (5)	0 (0)	1 (5)	0.29
Recovered	14 (28)	18 (37)	15 (31)	2 (4)	0 (0)	
*Statement 2:* I would be willing for my child to be included in a clinical trial of these two commonly used fluids **without the trial being**, **explained to me beforehand**.						
Bereaved	3 (17)	2 (11)	0 (0)	7 (39)	6 (33)	1.00
Recovered	4 (8)	5 (10)	7 (15)	21 (44)	11 (23)	
*Statement 3:* I would like to be told that my child was being included in the trial and to be asked for consent for their information to be included in the trial as soon as their condition stabilised.						
Bereaved	0 (0)	2 (11)	2 (11)	8 (45)	6 (33)	0.68
Recovered	0 (0)	6 (12)	10 (20)	17 (35)	16 (33)	
*Statement 4:* I would **not want** to be told about the trial at any time, as long as both fluids are considered safe.						
Bereaved	6 (33)	6 (33)	2 (11)	3 (17)	1 (6)	0.10
Recovered	7 (14)	18 (37)	6 (12)	7 (14)	11 (23)	

Figures are n(%). Missing responses: Question 1∶1 bereaved; Question 2∶1 bereaved and 1 recovered; Question 3∶1 bereaved; Question 4; 1 bereaved.

**Table 2:** Scenario B: If a child could not be resuscitated and unfortunately died in the emergency department we would want to tell the parents that their child had been given the fluid as part of a clinical trial of emergency treatments.

	Strongly disagree	Disagree	Neither agree nor disagree	Agree	Strongly agree	p-value
*Statement 5:* I think in the scenario described it would be better **not to tell** the bereaved parent/carer about the trial at any time.						
Bereaved	6 (33)	6 (33)	0 (0)	1 (6)	5 (28)	0.06
Recovered	7 (14)	11 (23)	3 (6)	8 (16)	20 (41)	
*Statement 6:* I think in the scenario described it would be better to include a child’s records in the trial **without immediately asking** the bereaved parent/carer, and seek their consent later at the most appropriate time.						
Bereaved	3 (17)	3 (17)	0 (0)	7 (39)	5 (27)	0.59
Recovered	3 (6)	13 (27)	10 (20)	16 (33)	7 (14)	
*Statement 7:* I think in the scenario described it would be better to tell the bereaved parent/carer about the trial and seek their consent **before** including their child’s records in the trial.						
Bereaved	3 (16)	5 (27)	1 (5)	5 (26)	5 (26)	0.19
Recovered	11 (22)	17 (35)	2 (4)	14 (29)	5 (10)	

Figures are n(%). Missing responses: Question 5∶1 bereaved; Question 6∶1 bereaved.

### Discomfort with Medical Uncertainty

In their free text responses parents commented on the need for medical research.

“I understand that you are only trying to better the elimination of meningitis, and in order to do so you have to carry out these trials” [6R]

and made altruistic statements indicating that they wanted to contribute to such research and the ‘trust’ they needed to place with the clinical team.

“A parent in this situation has to trust the doctor who is making the decisions, just as one would rely on a fireman to put out the flames in one’s house” [6B].

However, parents commented that mention of a trial in the emergency situation would have *“alarmed”* them [8R]. They pointed to the powerful and frightening connotations the word ‘trial’ held: “*the word ‘trial’ is a scary term – I don’t think parents would be happy to have something ‘tried’ out on their child*” [13R]; “*an experiment with my child’s life*” [9R].

Some parents indicated that they equated medical uncertainty with lack of expertise of the clinical team:

“If we thought […] that the staff did not fully know what they were doing or administering fluids they did not know would work, we would have been horrified” [3B].

Concerns were also raised that the approach about a trial would raise questions in their minds about whether a child’s survival was a clinical team’s primary concern: “‘*playing’ with that person’s life by conducting a trial*” [3B]; *“we wanted to see people doing things to help my son […] mentioning trials may have alarmed us…”* [8R] and implied *“that there might be a better option for your child”* [5R].

Parents’ clearly wanted to convey the depth of their concerns. Importantly, however, only 6% (3/67) did not want their child to be involved in the trial under any circumstances (Table 1: statement 1). Of these parents, two wrote qualitative responses indicating that they found it difficult to accept the medical uncertainty that they associated with the trial. Although the responses grouped by bereavement status were not statistically significant, 90% of bereaved parents compared with 65% of recovered parents would want their child to be included in the trial (p = 0.29). Those in the recovered group gave a greater proportion of responses in the ‘neither agree nor disagree’ category compared to bereaved responders (31% compared to 5%).

### Is it Acceptable to Defer Consent?

Statement 2 explored whether parents would be willing for their child to be included in the trial without the trial being explained to them beforehand.

Sixty-eight percent (45/66) responded positively. Again a higher percentage of those in the recovered group responded in the neutral ‘neither agree nor disagree’ category (15% compared to 0%).

70% (47/67) of parents wanted to be informed that their child had been included in the trial and to be asked for consent as soon as their child’s condition had stabilized (statement 3). 33% (22/67) did not want to be told about the trial at any time as long as both treatments were considered safe (statement 4); 55% (37/67) disagreed with this. While there were no statistically significant differences between the groups (p = 0.1), a higher proportion of bereaved responders (33% compared with 14%) felt strongly that they would want to be told about the trial at some time. At the opposite extreme, 6% (1/18) bereaved and 23% (11/49) of parents of recovered children would not want to be told about the trial at any time.

In the free-text responses one parent of a child who recovered expressed total disagreement with the concept of deferred consent:

“In that life and death scenario (which we have been in) you would not want your child’s life to be in a trial. Consent must and should be granted by parent before any trial is started.” [21R]

Some other parents wrote of the adverse emotional impact that deferred consent would have: “*if staff had proceeded without asking me first I would have been very annoyed”* [16R], while others emphasised the importance of being honest with parents or implied that deferred consent involved concealing information *“best not to hide anything from the parents at all”* [14R]. Several wrote that the trial would be ‘blamed’ if the child died or had a poor outcome or indicated that they would seek legal redress:

“I would have sued anyone that moved if he had died, being involved in a clinical trial that we knew nothing about” [12R].

While acknowledging that many difficult decisions are unavoidable in these circumstances, one bereaved parent remarked that “*any explanation and consent is better than none”* [1B].

Of the 14 parents who disagreed with their child being involved without the trial being explained beforehand (statement 2) all agreed with being told their child was being included in the trial and asked for consent once the child’s condition had stabilised (statement 3).

### Seeking Deferred Consent when a Child has Died

Scenario B considered the profoundly difficult situation in which a child enrolled in the trial had died prior to the trial being discussed with the parents and consent sought. While 57% of parents of children who recovered felt that it would be better not to tell the bereaved parent about the trial at any time, only 34% of the bereaved parents felt this way. The majority of bereaved parents (66%) disagreed with this statement (statement 5, Table 2).

Qualitative responses from the parents who did not want to be told about the trial at any time indicated that they believed an explanation of the trial was unnecessary. Clinical equipoise and the safety and current use of the two treatments featured prominently in some parents’ justifications of non-disclosure:

“My understanding is that both treatments are currently used and that there is genuinely no established right or wrong. Therefore I don’t think the parents would need to know. Our son died in PICU [Paediatric Intensive Care Unit] […] and the last thing I would have wanted to be told was that my son was in a trial […] I don’t feel in a situation like this it is necessary or desirable to get consent”. [10B]

“On the basis that these two solutions are both used and considered safe and presumably she could have conceivably received either anyway, for me, knowing of the trial would have led to confusion and questioning which would have been pointless, negative and detrimental […] the trial is simply recording the results of her treatment, I would be happy to be ignorant of this.” [19B]

Parents’ comments were motivated by a wish to avoid adding to the distress of grieving parents. They wrote of how, in the aftermath of a child’s death, parents were intensely vulnerable and it was hard for them to be rational. In this context information about a trial confronted them with knowledge that could be destructive and exacerbate a parents grief.

Other parents pointed to the disruption for hospitals and potential for litigation that disclosure could bring:

“They will not “hear” or understand the fact both fluids are in fact safe […] grief makes people irrational […] people would consult legal advisors, and in general it would cause more distress.” [17R]

Of the six bereaved parents whose free text responses indicated they opposed disclosure, in their subsequent comments about the need to carefully time disclosure, three of these parents implied that being told about the trial at some point might not be completely beyond their contemplation. The remaining three bereaved parents seemed resolute that they would not want to be told about the trial at any point. Parents who were in favour of disclosure also acknowledged the difficulties that could arise from telling parents about the trial in such an emotionally charged situation and several sympathised with practitioners and the complexities they would encounter in disclosing details of the trial.

### Timing and Managing the Approach for Deferred Consent

Statements 6 and 7 considered the inclusion of the child’s records in the research and the timing of consent. Of the 18 bereaved parents who responded to the survey eight agreed that it was better to include the child’s records in the trial without immediately obtaining consent (statement 6) and disagreed with seeking consent before including the child’s records (statement 7).

Question 8 invited parents to comment about what would be the most appropriate time for practitioners to explain about the trial and ask for consent if a child died.

There was no overall consensus regarding the ‘right time to tell’ among the 34 parents who gave free text responses (12 bereaved; 22 recovered), However, recovered parents were rather more precise in setting a time frame for disclosure compared to their bereaved counterparts. For example: “immediately or as soon as possible”[12R]; “as soon as possible after death” [16R]; “at the time of post mortem” [4R]; “after 3 days once the initial shock has receded” [7R]; “maybe 2 weeks” [20R].

Bereaved parents were less prescriptive: “*as soon as it’s appropriate”* [11B]*;* “*when not too upset to understand”* [15B]*; “[when] you’re not too upset, too much you can’t understand”* [15B]. One bereaved parent described how nearly six weeks had passed after her child’s death before she could “face” discussing what had happened with the consultant [10B]. Another suggested that trial discussion could be conducted alongside those about organ donation, as she felt that she had been able to “*take in*” information at that point [2B], while another wrote of how she had valued a home-visit from a Community Nurse Specialist who had explained about her child’s death in a way she found helpful and sensitive, adding that such a visit “*a few days after bereavement*” [18B] could also lend itself to discussion about the trial.

Question 9 asked ‘What do you think would be the best way to explain the trial and ask for consent?’ and invited parents to select one of three options. Forty nine (13 bereaved, 36 recovered) responded. Fifteen parents (6 bereaved, 9 recovered) selected the option with the ‘doctor or nurse’, three (recovered) selected ‘written information’, 28 (7 bereaved, 21 recovered) indicated ‘both consultation and written information’. Three parents indicated ‘neither of these’.

Parents’ additional free text responses indicated they wanted calm and concise information giving with time to ask questions and the opportunity for follow-up. Implicit in their responses was the need for a caring, flexible and responsive approach to communication.

## Discussion

We investigated the acceptability of deferred consent in a paediatric emergency setting. Results indicated that deferred consent is acceptable to the majority. Although the survey was not powered to detect statistically significant differences between the groups, parents of children who recovered tended to indicate neutral opinions more frequently. The groups had differing views regarding disclosure of the trial in the event a child died before the trial could be discussed. Two thirds of bereaved parents opposed non-disclosure of the trial compared to just over a third of parents whose child recovered. While the sample of bereaved parents was small, in drawing conclusions about trial disclosure in the context of a child’s death, it could be argued that greater weight should be given to the responses of bereaved parents. However, there was a sizeable group of bereaved parents at each end of the spectrum which reflects the variety inherent in the experience of bereavement and the difficulty in developing a policy that will suit the population concerned. Developing the means to respond to this variety is the challenge ahead.

While the response rate to this survey was low it is similar to other postal surveys [17], however non-response bias is unlikely to be an issue with the percentage of bereaved parents being similar between responders and non-responders. Cross tabulations of responses between questions indicated the consistency of responses suggesting that the responders had understood the survey. Parents were able to allocate time to discuss, reflect upon and complete this survey in their own homes. The potential for misinterpreting information could be exacerbated when encountering these issues in the crisis of emergency treatment of a child. It is also possible that parents’ interpretation of the same trial could have been very different if they had encountered it in a real-life context in which they had a relationship with the clinical team conducting the trial. The meanings parents take away from face-to-face discussions about a trial with caring, committed and compassionate clinicians are likely to differ from the ones that they had been left with by a description, received through the post, of a trial being conducted by researchers they had never met before. Paediatric clinical trials using deferred consent should conduct qualitative research on the acceptability of the process during the trial conduct to inform future trials.

The parents in our survey all had experience of having a critically ill child and this obliges us to take careful account of their perspectives, yet few if any are likely to have had experience of being approached about their child’s participation in a trial in the emergency setting and it is difficult for anyone to anticipate how they might respond in such a situation.

In a trial comparing similar interventions to the trial proposed here, Maitland et al [4], present a modified deferred consent model in the treatment of children requiring fluid resuscitation in hospitals in three malaria-endemic African countries. This involved taking verbal assent from parents at the point of enrolment, with full written consent being sought after stabilising the child; but for parents of children who died prior to full written consent, ethical permission was received to waiver full consent. In indicating that the majority of parents would be willing for their child to be included in the trial without the trial being explained beforehand and the majority of bereaved parents indicating that they would want to be informed, our survey’s findings do not support the model used by Maitland et al [4]. The impact of any assent process on parents, clinicians and the patient in terms of the assent process delaying critical treatment should be considered.

Our findings indicate that deferred consent is generally acceptable in principle to most parents, but also that disclosure is not something that can be written into ‘one size fits all’ protocols. The death of a child during a trial in which deferred consent has been used is a uniquely difficult situation, and further evidence and ethical guidelines are needed regarding the appropriate handling of this situation.

Communication should be flexible and responsive to the needs of individual parents. In this sense – and it’s by no means a new concept – communication about trials needs to be practiced as a process of individualised care, rather than as routinised procedures. Parents value the *quality of the relationship* with the expert care team and this will shape their interpretations of the trial [18]. However, practitioners of course need the time, training and experience to ‘read and respond’ to individual circumstance.

Parents responses indicated the particularly profound complexities involved in seeking deferred consent after a child’s death and the potential to exacerbate a parent’s grief, a risk that some thought unnecessary given that both trial treatments were in current use. Risk proportionate approaches are being applied to clinical trials governance [19] and questions about how this could be extended to models of consent should be raised.

## Supporting Information

Appendix S1 **Postal survey.**
(DOC)

## References

[1] Stephenson T (2006). The Medicines for Children Agenda in the UK.. Br J Clin Pharmacol.

[2] regulations and administrative provisions of the Member States relating to the implementation of good clinical practice in the conduct of clinical trials on medicinal products for human use. Official Journal of the European Communities L 121, 01/05/2001 P. 0034–0044

[3] Legislation.gov.uk website. Medicines for Human Use (Clinical Trials) Regulations.. http://www.opsi.gov.uk/si/si2004/20041031.

[4] Maitland K, Molyneux S, Boga M, Kiguli S, Lang T (2011). Use of deferred consent for severely ill children in a multi-centre phase III trial.. Trials.

[5] Legislation.gov.uk website.The Medicines for Human Use (Clinical Trials) Amendment (No. 2) Regulations (2006). http://www.opsi.gov.uk/SI/si2006/20062984.htm.

[6] Legislation.gov.uk website.The Medicines for Human Use (Clinical Trials) and Blood Safety and Quality (Amendment) Regulations 2008 941.. http://www.legislation.gov.uk/uksi/2008/941/pdfs/uksi_20080941_en.pdf.

[7] Federal register (1996). Protection of human subjects; informed consent-FDA. Final rule..

[8] Morrison C, Horwitz I, Carrick M (2009). Ethical and legal issues in emergency research; Barriers to conducting prospective randomized trials in an emergency setting.. Journal of Surgical Research.

[9] Jansen T, Kompanje E, Druml C, Menon D, Wiedermann C (2007). Deferred consent in emergency intensive care research: what if the patient dies early? Use the data or not?. Intensive Care Med.

[10] Jansen TC, Bakker J, Kompanje EJ (2010). Inability to obtain deferred consent due to early death in emergency research: effect on validity of clinical trial results.. Intensive Care Med.

[11] Nichol G, Powell J, van Ottingham L, Maier R, Rea T (2006). Consent in resuscitation trials: Benefit or harm for patients and society?. Resuscitation.

[12] National Collaborating Centre for Women’s and Children’s Health (2010). Bacterial meningitis and meningococcal septicaemia in children..

[13] Braun V, Clarke V (2006). Using thematic analysis in psychology.. Qualitative Research in Psychology.

[14] Fereday J, Muir-Cochrane E (2006). Demonstrating Rigor Using Thematic Analysis: A Hybrid Approach of Inductive and Deductive Coding and Theme Development.. International Journal of Qualitative Methods.

[15] Giacomini M, Ward D (2000). Users’ guides to the medical literature XXIII. Are the results of the study valid?. JAMA.

[16] Spencer L, Ritchie J, Lewis J, Dillon L (2003). Quality in qualitative evaluation: A Framework for Assessing Research Evidence..

[17] Culbert A, Davis D (2005). Parental preferences for neonatal resuscitation research consent: a pilot study.. Journal of medical ethics.

[18] Shilling V, Williamson P, Hickey H, Sowden E, Beresford M (2011). Communication about children’s clinical trials as observed and experienced: qualitative study of parents and practitioners.. PLoS One.

[19] Smyth R (2011). A risk adapted approach to the governance of clinical trials.. BMJ 343,.

