# Supplementary material for: What Parents of Children Who Have Received Emergency Care Think about Deferring Consent in Randomised Trials of Emergency Treatments: Postal Survey
Source: PLoS One. 2012 May 7;7(5):e35982. doi: 10.1371/journal.pone.0035982 (PMC3346812; doi:10.1371/journal.pone.0035982)
Supplement: Appendix S1 — Postal survey. (DOC) [file pone.0035982.s001.doc]

**Appendix S1: Postal survey**

**“SOAR” (Saline Or Albumin for Resuscitation) - a double blind, randomised controlled trial comparing the effectiveness of 0.9% saline solution with 4.5% human albumin solution for resuscitation in children with presumed severe sepsis.**

**Why do we want to carry out a trial?**

Each year several thousand very sick children with severe infections (sepsis) attend accident and emergency departments in the UK. Some of these children are too sick to be saved and sadly die in the emergency department or in the paediatric intensive care unit (PICU). It is estimated that of the 2000 children each year who are admitted to PICUs, 400 will not recover.

One of the main causes of death in these children is the impact the infection has on their blood circulation. Children with serious circulation problems need to be given immediate treatment (emergency resuscitation) and large amounts of fluid to improve their blood flow, but a major question for doctors is that no one knows for certain which fluid is the most effective for children. Some doctors think salt water (saline) is best and others think Human Albumin Solution (HAS) is best^1^. Both types of fluid are widely used outside of clinical trials and are known to be safe. Saline is the one used most frequently, mainly because it is more readily available.

It is possible that one of these fluids is better than the other for treating circulation problems in children with severe sepsis, and will help more children to survive. The **only** way to properly answer the question about which fluid is the most effective is to carry out a special type of medical research study called a randomised controlled trial. We are planning to conduct just such a trial.

**How do we intend to carry out the trial?**

As in all high quality trials, the fluid treatment given to each child within the trial will be chosen by chance (similar to tossing a coin). This is the only way to conduct the trial properly and avoid the results being biased. Half the children in the trial will receive saline, and the other half HAS.

It is also important that neither the staff caring for the children, nor the child or their parents/carers know which treatment they are receiving (although if the doctors need to find out for a medical reason they can do so). This is called “double blind”. It’s to make sure that opinions and decisions are not swayed or biased by knowing whether the child is receiving saline or HAS.

Both treatments will be held in each of the Accident and Emergency departments and will be packaged so that each pack (containing either saline or HAS) will look exactly the same. When a child needs emergency fluid treatment the doctor or nurse will pick up the next available treatment pack. The packs will be kept together so the doctor or nurse will not know whether they have picked up saline or HAS, but all of the treatment packs will carry a trial code so they could find out which fluid was given if necessary. We are planning the trial in this way so that it is double blind and to ensure there will be no delays in treating any child. All children in the trial will then be closely monitored during their time in hospital and for up to 6 months afterwards.

At the end of the trial, the results from the two groups of children (i.e. the group who received saline and the group who received HAS) will be compared. We hope this will tell us which of these two fluids can best help children survive sepsis and go on to lead normal, healthy lives. After the trial is finished, the findings will be incorporated into treatment guidelines for doctors across the UK so they know how best to treat children in the future.

This trial will take place in emergency departments, children's wards and PICUs throughout the UK, by a network of doctors and research staff who are very experienced in carrying out research studies in seriously ill children. Before we are able to start the trial we will need approval from an independent ethics committee and from the UK regulatory authorities.

**Why this trial is difficult to do**

Usually before a child can join a trial it is necessary to obtain the informed consent of the parent or carer. This usually involves a detailed discussion about the risks and benefits of the trial treatments and giving the parents several hours to decide if they want their child to be included. Unfortunately, because the trial we intend to do involves giving emergency treatment, where any delay in starting treatment can increase the risk of the child dying, there will not usually be time to properly inform the parents/carers about the trial and ask for their written agreement before starting treatment.

Therefore, because of the emergency nature of the treatment and our wish to avoid any delay in giving the fluid treatment, **we do not intend to ask parents/carers for their consent before entering their child into the trial.**

In these circumstances there are special guidelines for researchers to follow called a **‘deferred consent process’**. This means that the fluid treatment (either saline or HAS) will be given to a child as quickly as possible without asking for parental consent. However, as soon as the child’s condition has been stabilised the researchers will ask for consent from the child’s parent/carer (and from the child where appropriate). In this case, we will ask for consent for the child to continue in the trial and also to include the relevant details of their medical records in the trial. At this point the parents/carers may refuse. If the parents/carers refused, the care of the child will not be affected, but any further treatment received would not be given as part of the trial and none of the child’s information will be included in it.

Without this trial we may never know which type of fluid treatment is best for children, but we are also very aware of the sensitivities surrounding our research plans and the fact that it will not be possible to obtain informed consent before treating a child who needs emergency resuscitation. We want to try our best to make this trial as acceptable to parents/patients as possible.

Because of your past experience of meningitis or septicaemia, we believe that your views will help us enormously to ensure that the way we conduct the trial is as good as it can possibly be. We would be very grateful to know your opinion of the two scenarios and questions on the next pages. It’s important to mention that there are no ‘right’ or ‘wrong’ answers to these – it is your views that are important to us.

**Scenario A:** If a child of mine had a serious infection and needed emergency fluid treatment:

1. I would **not want** my child to be included in a clinical trial of these two commonly used fluids under any circumstances. (*please circle the response you prefer*)

Strongly disagree/ Disagree/ Neither agree nor disagree/ Agree/ Strongly agree

1. I would be willing for my child to be included in a clinical trial of these two commonly used fluids, **without the trial being explained to me beforehand.** (*please circle the response you prefer*)

Strongly disagree/ Disagree/ Neither agree nor disagree/ Agree/ Strongly agree

1. I would like to be told that my child was being included in the trial and to be asked for consent for their information to be included in the trial as soon as their condition stabilised. (*please circle the response you prefer*)

Strongly disagree/ Disagree/ Neither agree nor disagree/ Agree/ Strongly agree

1. I would **not want** to be told about the trial at any time, as long as both fluids are considered safe. (*please circle the response you prefer*)

Strongly disagree/ Disagree/ Neither agree nor disagree/ Agree/ Strongly agree

**Scenario B:** If a child could not be resuscitated and unfortunately died in the emergency department we would want to tell the parents that their child had been given the fluid as part of a clinical trial of emergency treatments. However, we are not sure whether this is the right thing to do, or how we should do it.

We would like to know how you feel about whether, at what time and how we should explain about the trial and the fluid treatments in these very difficult circumstances. We would also like to know how to seek consent to include a child’s records in the trial.

1. I think in the scenario described it would be better **not to tell** the bereaved parent/carer about the trial at any time. (*please circle the response you prefer*)

Strongly disagree/ Disagree/ Neither agree nor disagree/ Agree/ Strongly agree

1. I think in the scenario described it would be better to include a child’s records in the trial **without immediately asking** the bereaved parent/ carer, and seek their consent later at the most appropriate time. (*please circle the response you prefer*)

Strongly disagree/ Disagree/ Neither agree nor disagree/ Agree/ Strongly agree

1. I think in the scenario described it would be better to tell the bereaved parent/carer about the trial and seek their consent **before** including their child’s records in the trial. (*please circle the response you prefer)*

Strongly disagree/ Disagree/ Neither agree nor disagree/ Agree/ Strongly agree

If your answer to **question 7** was one of “Neither agree nor disagree”, “Agree” or

“Strongly agree”, we would be grateful if you could also answer the following questions:

1. When do you think would be the most appropriate time to explain about the trial and ask for consent?
2. What do you think would be the best way to explain about the trial and ask for consent? (*please circle the response you prefer*)

Consultation with the doctor and/or nurse/ Written information/ Both of these/

Neither of these/ Other (please state below)

We understand that these questions may have been very difficult for you to answer and are very grateful for your help. If you have any other comments on what we are trying to do, especially about the consent issues, we would welcome them, either written in the space below, or at the address on the accompanying letter, by email or post.
